# Proline/alanine-rich sequence (PAS) polypeptides as an alternative to PEG precipitants for protein crystallization

**DOI:** 10.1107/S2053230X20008328

**Published:** 2020-07-01

**Authors:** André Schiefner, Rebecca Walser, Michaela Gebauer, Arne Skerra

**Affiliations:** a Lehrstuhl für Biologische Chemie, Technische Universität München, 85354 Freising, Germany; b XL-protein GmbH, Lise-Meitner-Strasse 30, 85354 Freising, Germany

**Keywords:** disordered polypeptide, PASylation, protein precipitant, polyamino acid, polyethylene glycol, proline/alanine-rich sequence, protein crystallization

## Abstract

Proline/alanine-rich sequence (PAS) polypeptides represent a novel class of biosynthetic polymers comprising repetitive sequences of the small proteinogenic amino acids l-proline, l-alanine and/or l-serine. PAS polymers are strongly hydrophilic and highly soluble in water, where they exhibit a natively disordered conformation without any detectable secondary or tertiary structure, similar to polyethylene glycol (PEG), which constitutes the most widely applied precipitant for protein crystallization to date. To investigate the potential of PAS polymers for structural studies by X-ray crystallography, two proteins that were successfully crystallized using PEG in the past, hen egg-white lysozyme and the *Fragaria × ananassa* *O*-methyltransferase, were subjected to crystallization screens with a 200-residue PAS polypeptide. The PAS polymer was applied as a precipitant using a vapor-diffusion setup that allowed individual optimization of the precipitant concentration in the droplet in the reservoir. As a result, crystals of both proteins showing high diffraction quality were obtained using the PAS precipitant. The genetic definition and precise macromolecular composition of PAS polymers, both in sequence and in length, distinguish them from all natural and synthetic polymers that have been utilized for protein crystallization so far, including PEG, and facilitate their adaptation for future applications. Thus, PAS polymers offer potential as novel precipitants for biomolecular crystallography.

## Abstract 2

The suitability of a PAS polypeptide as a precipitant to grow protein crystals with high X-ray diffraction quality has been demonstrated, adding this novel class of biosynthetic polymers to the toolset for protein crystallography as an alternative to polyethylene glycol.

## 1. Introduction   

Crystallization of proteins and nucleic acids still remains the major limiting factor for structural analysis using X-ray diffraction. Apart from sample quality (Dale *et al.*, 2003 ▸), various factors play a role, in particular the nature of the precipitating agent as well as the ionic strength, the pH and the temperature, the influences of which are difficult to predict owing to the structural complexity of biological macromolecules (McPherson & Gavira, 2014 ▸). To compensate for this lack of information, automated submicrolitre sparse-matrix screening methods based on the empirical knowledge of previously successful conditions have become the state of the art in protein crystallography (Stewart & Mueller-Dieckmann, 2014 ▸). In contrast, the search for novel types of precipitants has diminished significantly in recent years.

The precipitants utilized in protein crystallization are generally classified into inorganic salts, organic salts, organic solvents and water-soluble organic polymers. Polyethylene glycol (PEG) was the first such polymer to be introduced into protein crystallization, enabling the X-ray structure determination of deoxyhemoglobin A from crystals grown in a PEG-containing buffer (Ward *et al.*, 1975 ▸). Just one year later, the general utility of PEG for protein crystallization was proposed (McPherson, 1976 ▸). While a range of average molecular PEG masses from 200 to 20 000 Da proved to be suitable for protein crystallization, PEG masses of 2000–8000 Da are most frequently applied in commercially available screens. Meanwhile, PEG monomethyl ethers (PEG MMEs), which exhibit very similar precipitation properties, have also been adopted in protein crystallization (Brzozowski & Tolley, 1994 ▸). Notably, about half of the crystallization conditions reported in the Protein Data Bank (PDB) comprise some kind of PEG component (Peat *et al.*, 2005 ▸).

Based on the notion that high-molecular-weight polymers such as PEG induce protein precipitation/crystallization by increasing macromolecular crowding (Majeed *et al.*, 2003 ▸), several other types of synthetic organic polymers have been investigated for their suitability as precipitating agents in protein crystallography over the years: (i) Jeffamine (polyetheramines) as well as polyethylene imine (Cudney *et al.*, 1994 ▸), (ii) polyacrylate, polyvinyl pyrrolidone, polyvinyl alcohol, polypropylene glycol and PEG dimethyl ether (Patel *et al.*, 1995 ▸), (iii) pentaerythritol propoxylate (Gulick *et al.*, 2002 ▸), (iv) di(polyethylene glycol) adipate (Kolenko *et al.*, 2009 ▸) and (v) acrylic acid/maleic acid copolymers, glycerol ethoxylate, polyacryl amide and vinylpyrrolidone/vinylimidazole copolymers (Grimm *et al.*, 2010 ▸). Furthermore, natural as well as semisynthetic polymers have been applied, such as carboxymethyl cellulose (Patel *et al.*, 1995 ▸), hydroxypropyl methylcellulose (Grimm *et al.*, 2010 ▸), poly-γ-glutamic acid (PGA) and PGA–glucosamine conjugates (Hu *et al.*, 2008 ▸).

**Figure 1:**
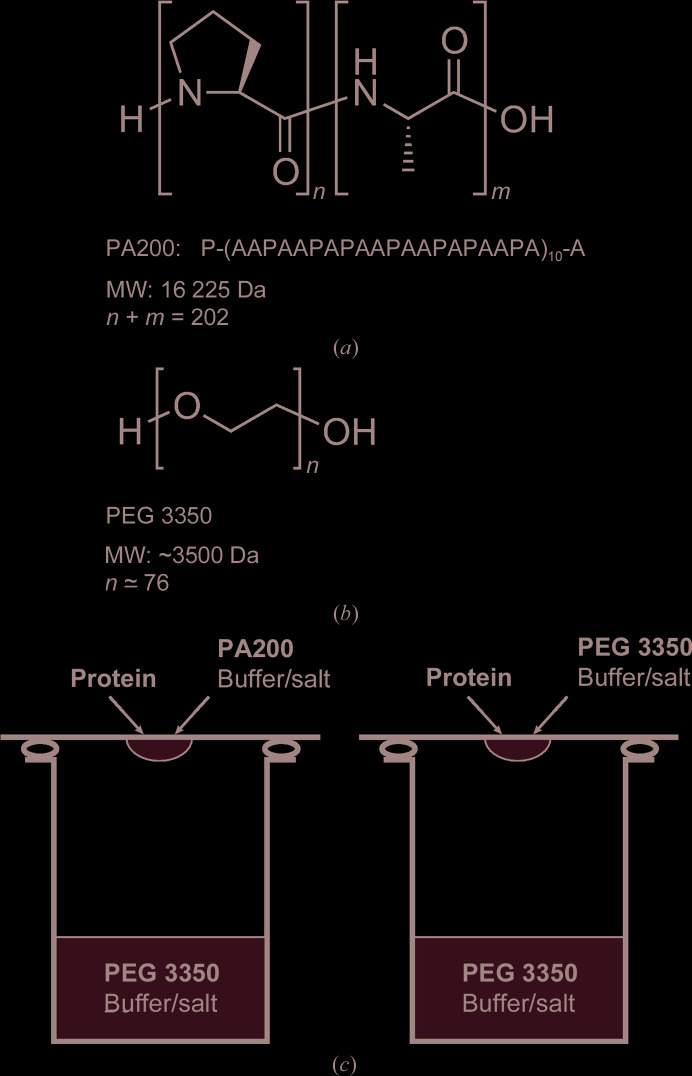
Comparison of PAS polypeptides with conventional PEG precipitants. Chemical constitution of PA200 (*a*) *versus* PEG 3350 (*b*). (*c*) Side-by-side vapor-diffusion experiment using PA200 and PEG 3350 as precipitants for protein crystallization, with PEG 3350 serving as the common hygroscopic polymer solute in the buffer reservoir.

However, the use of structurally disordered polypeptides as precipitants has not been reported to date. Proline/alanine-rich sequences (PAS) represent a novel class of biodegradable biopolymers that are currently under development as a biological alternative to PEG for extension of the plasma half-life of therapeutic proteins (Gebauer & Skerra, 2018 ▸; Schlapschy *et al.*, 2013 ▸). PAS polymers, which consist of the small uncharged proteinogenic l-amino acids proline, alanine and/or serine (Fig. 1 ▸), exhibit physicochemical properties that are surprisingly similar to those of PEG. Notably, PAS polypeptides of comparable mass show an increased hydrophilicity and hydrodynamic volume but a lower viscosity than PEGs (Breibeck & Skerra, 2018 ▸). Moreover, PAS polymers have no net charge and do not affect the pH of aqueous solutions. Intriguingly, as genetically encoded recombinant polypeptides they possess a precisely defined composition and length, which differentiates these biosynthetic macromolecules from all of the other polymers mentioned above.

Thus, we wondered whether PAS polypeptides may also show potential as precipitants for protein crystallization. In order to investigate their applicability, we performed vapor-diffusion experiments with two previously crystallized model proteins, hen egg-white lysozyme (HEL; UniProtKB P00698) and the *Fragaria × ananassa* *O*-methyltransferase (FaOMT; Wein *et al.*, 2002 ▸), using a biosynthetic 200-residue Pro/Ala polymer (PA200) as a precipitant.

## 2. Materials and methods   

HEL was purchased from AppliChem (Darmstadt, Germany) and dissolved at 30 mg ml^−1^ in 10 m*M* sodium acetate pH 4.5. FaOMT was purified as described elsewhere (Schiefner *et al.*, in preparation) and concentrated to 14.4 mg ml^−1^ in 150 m*M* NaCl, 20 m*M* HEPES–NaOH pH 7.5, 2 m*M* β-mercapto­ethanol, 0.02%(*w*/*v*) sodium azide with the addition of 2 m*M* *S*-adenosylmethionine (SAM). Both enzymes were known to crystallize in the presence of PEG 3350 as a precipitant: in 20%(*w*/*v*) PEG 3350, 0.2 *M* NaCl, 0.1 *M* sodium acetate pH 4.5 for HEL (Beck *et al.*, 2008 ▸; Luo, 2016 ▸) and 20%(*w*/*v*) PEG 3350, 0.2 *M* MgCl_2_, 0.1 *M* HEPES–NaOH pH 7.0 for FaOMT–SAM. In the present study, PEG 3350 was substituted by suitable concentrations of the Pro/Ala polymer using the same buffer conditions.

The recombinant monodisperse 200-residue Pro/Ala polymer (PA200) was produced in *Escherichia coli* and purified to homogeneity according to published procedures (Binder *et al.*, 2017 ▸; Breibeck & Skerra, 2018 ▸) and was lyophilized from 0.3 m*M* acetic acid (Fig. 1 ▸). PA200 was dissolved in distilled water supplemented with 0.02%(*w*/*v*) sodium azide to yield a 50%(*w*/*v*) stock solution.

The precipitation efficiency of PA200 was investigated by mixing 1 µl of a 6.25, 12.5, 25 or 50%(*w*/*v*) PA200 solution with 1 µl protein solution to yield initial PA200 concentrations of up to 25%(*w*/*v*). Crystallization of HEL and FaOMT was performed by hanging-drop vapor diffusion in Crystalgen SuperClear plates (Jena Bioscience, Jena, Germany) by mixing 1 µl crystallization solution (precipitant plus buffer) with 1 µl protein solution. The crystallization solution consisted of 6.25 or 12.5%(*w*/*v*) PA200 with 0.2 *M* NaCl, 0.1 *M* sodium acetate pH 4.5 or with 0.2 *M* MgCl_2_, 0.1 *M* HEPES–NaOH pH 7.0 for HEL or FaOMT, respectively. In order to keep the consumption of the Pro/Ala polymer low, the 1 ml reservoir solutions contained stepwise increasing concentrations of PEG 3350 instead of PA200 (Figs. 1 ▸ and 2 ▸). To confirm that the use of PEG in the reservoir, serving to withdraw the water from the crystallization droplets via vapor diffusion, is a viable approach in our setup, the same experiment was performed using 6.25 or 12.5%(*w*/*v*) PEG 3350 (instead of PA200) in the crystallization droplet (Figs. 1 ▸ and 3 ▸).

Prior to flash-cooling, the crystals were quickly transferred into a cryoprotectant solution consisting of 12.5%(*w*/*v*) PA200, 20%(*v*/*v*) ethylene glycol, 0.2 *M* NaCl, 0.1 *M* sodium acetate pH 4.5 for the HEL crystals and 12.5%(*w*/*v*) PA200, 20%(*v*/*v*) ethylene glycol, 0.2 *M* MgCl_2_, 0.1 *M* HEPES–NaOH pH 7.0 for the FaOMT crystals. X-ray diffraction data were collected on beamline 14.2 of BESSY at the Helmholtz-Zentrum Berlin (Mueller *et al.*, 2012 ▸) and were reduced using the *XDS* package (Kabsch, 2010 ▸; Table 1 ▸). The crystal structures were solved by molecular replacement with *Phaser* (McCoy *et al.*, 2007 ▸) using previously deposited coordinate sets for HEL (PDB entry 5t3f; Luo, 2016 ▸) and FaOMT (PDB entry 6i71; Schiefner *et al.*, in preparation) as search models. Model building and refinement were performed with *Coot* (Emsley *et al.*, 2010 ▸) and *REFMAC*5 (Murshudov *et al.*, 2011 ▸), respectively (Table 1 ▸). The coordinates and structure factors for the crystal structures of HEL and FaOMT determined with the help of the Pro/Ala polymer have been deposited in the PDB with accession codes 6yjw and 6yjx, respectively.

## 3. Results and discussion   

In contrast to the polydisperse synthetic or semisynthetic organic polymers used for protein crystallization to date, in particular PEG (Gaberc-Porekar *et al.*, 2008 ▸), PAS polypeptides exhibit a genetically defined length and sequence, which can be tuned according to custom needs. Generally, PAS polypeptides constitute highly water-soluble biosynthetic polymers (Breibeck & Skerra, 2018 ▸); for example, PA200 can be readily dissolved in aqueous buffers at concentrations of up to 50%(*w*/*v*), corresponding to 31 m*M*. The molecular mass of PA200 (16 225 Da) is 4.8-fold higher than the reference PEG polymer PEG 3350 applied in this study, whereas its length in a fully extended conformation exceeds that of PEG 3350 by a factor of just 2.6. While the number of hydrogen-bond acceptors per monomer unit (carbonyl groups in PA200 versus ether O atoms in PEG 3350) is identical for PA200 and PEG 3350, 65% of the monomer units in the Pro/Ala polymer, *i.e.* all of the alanine residues with their amide groups, also act as hydrogen-bond donors (Fig. 1 ▸). Prior to setting up protein crystallization experiments with PA200, its precipitation efficiency was examined by mixing HEL and FaOMT protein solutions with increasing PA200 concentrations (see Section 2). Both proteins started to precipitate at PA200 concentrations of around 6.25%(*w*/*v*).

**Figure 2:**
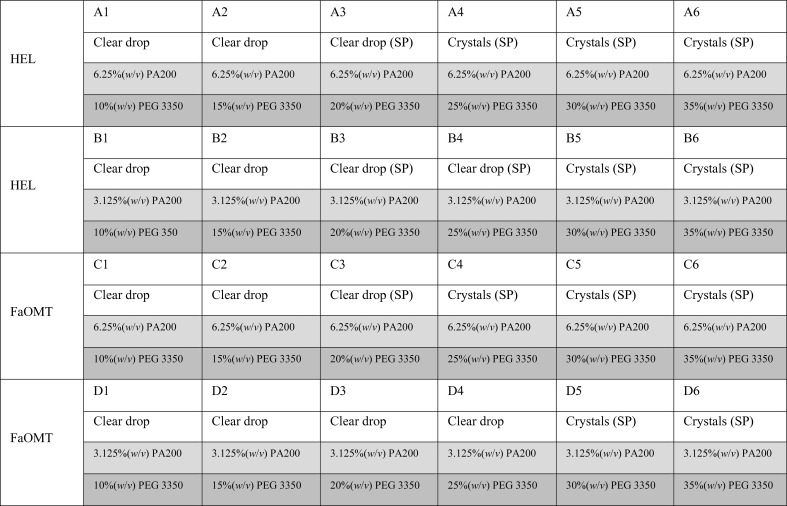
Crystallization-plate setup for PA200. Experimental observations are on a white background, starting precipitant concentrations in the droplets are shaded light gray and those in the reservoir are shaded gray. Spherulites (SP) appeared about two weeks after the crystals had formed.

Generally, in a vapor-diffusion experiment the protein sample is mixed with a certain volume of precipitant solution (typically in a 1:1 ratio) to yield a crystallization droplet. This droplet is then placed next to a larger volume of the precipitant solution, termed the reservoir, and is equilibrated in a sealed reaction vessel. Thereby, all solutes in the droplet are concentrated by way of vapor diffusion, considering that the initial precipitant/buffer concentration in the droplet is only 50% of that in the reservoir, until the supersaturation of the protein in the droplet is sufficient for nucleation and, ideally, crystal growth (McPherson & Gavira, 2014 ▸). To minimize the required amount of PA200 in our crystallization experiments for practical reasons, the classic setup was adapted such that PA200 was only applied in the crystallization droplet, whereas PEG 3350 was instead used in the reservoir solution. All other components, such as the buffer and salt, were identical in both solutions (Fig. 1 ▸ *c*). As it is difficult to estimate the concentration of PEG 3350 that is equivalent in terms of hygroscopy to a certain percentage of PA200, protein droplets with identical PA200 contents were equilibrated against varying concentrations of PEG 3350 as described below (see Fig. 2 ▸).

**Figure 3:**
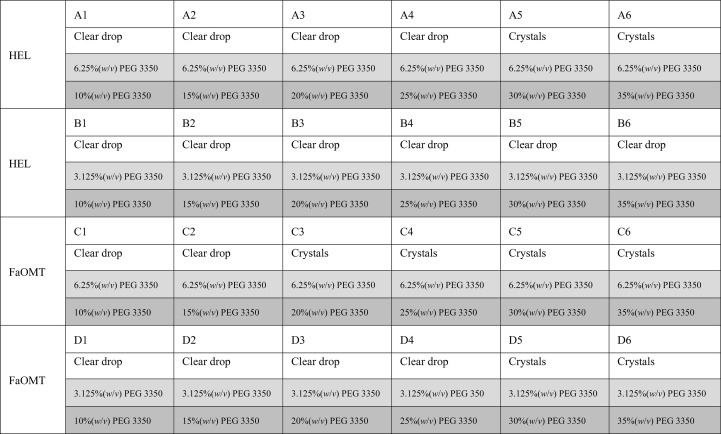
Crystallization-plate setup for PEG 3350. Experimental observations are on a white background, starting precipitant concentrations in the droplets are shaded light gray and those in the reservoir are shaded gray.

**Figure 4:**
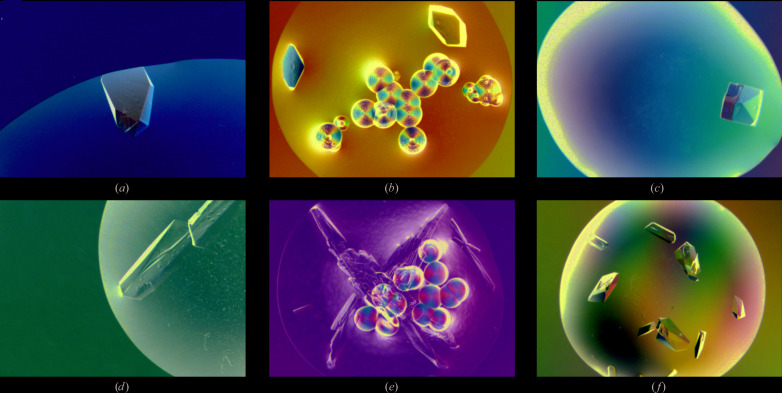
Protein crystallization using PA200 as a precipitant. (*a*) HEL and (*d*) FaOMT crystallized in the presence of 6.25%(*w*/*v*) PA200. For HEL (*b*) and FaOMT (*e*), spherulite formation was observed in the presence of 3.125 or 6.25%(*w*/*v*) PA200 approximately two weeks after the single crystals had appeared. HEL (*c*) and FaOMT (*f*) crystallized in the presence of 6.25%(*w*/*v*) PEG 3350 are shown for comparison (see Fig. 2 ▸).

In our setup, crystallization droplets were prepared by mixing 1 µl protein solution, at a concentration of 1.05 m*M* HEL or 182 µ*M* FaOMT, with 1 µl crystallization solution (precipitant plus buffer) containing 6.25 or 12.5%(*w*/*v*) PA200 to yield starting concentrations of the biosynthetic polymer of 3.125 or 6.25%(*w*/*v*) (193 or 385 µ*M*, respectively). These droplets were equilibrated against six different reservoir solutions containing 10, 15, 20, 25, 30 or 35%(*w*/*v*) PEG 3350 (Fig. 2 ▸). The first protein crystals appeared after one day for HEL as well as for FaOMT and at both of the applied PA200 concentrations. As expected, a lower PA200 concentration in the droplet required higher PEG 3350 concentrations in the reservoir to promote crystal growth: crystals of HEL and FaOMT in the presence of 3.125 or 6.25%(*w*/*v*) PA200 were observed at ≥30 and ≥25%(*w*/*v*) PEG 3350, respectively (Fig. 4 ▸). For comparison, the setup described above was repeated also using PEG 3350 in the crystallization droplet, applying the same mass concentrations of 3.125 or 6.25%(*w*/*v*), and again equilibrated against reservoir solutions containing 10–35%(*w*/*v*) PEG 3350 (Fig. 3 ▸). Crystals of both HEL and FaOMT were observed in a similar reservoir PEG concentration range as previously with the PA200 precipitant.

Interestingly, the morphology of the protein crystals grown in the presence of PA200 was very similar to those obtained with PEG 3350. It seemed that PEG led to the formation of crystals with slightly sharper edges and less macroscopic growth defects, which may, however, also have been a result of ordinary experimental variations. Notably, a considerable number of spherulites were observed with the PA200 precipitant beside the protein crystals after about two weeks (Fig. 4 ▸).

To demonstrate that the single crystals grown in the presence of PA200 were suitable for X-ray diffraction experiments, data sets were collected at the BESSY synchrotron source for both HEL and FaOMT and refined to resolutions of 1.2 and 2.1 Å, respectively. Both data sets showed reasonable statistics (Table 1 ▸). After molecular replacement and refinement, the structures were carefully inspected for residual electron density that might account for partially ordered PA200 molecules. However, no such density was observed, which suggests that the PAS precipitant did not specifically interact with the proteins, in accordance with experiences from other applications of PASylation technology (Binder & Skerra, 2017 ▸; Gebauer & Skerra, 2018 ▸). Otherwise, the atomic coordinates of the refined protein models were highly similar to the published crystal structures of HEL [PDB entry 5t3f; crystallized in 25%(*w*/*v*) PEG 3350, 50 m*M* citrate buffer pH 4.0; 1.45 Å resolution] and FaOMT [PDB entry 6i71; crystallized in 0.5–1 *M* ammonium sulfate, 1 *M* lithium sulfate, 0.1 *M* sodium malonate; 1.40 Å resolution], with r.m.s.d. values of 0.28 and 0.50 Å for 129 and 352 equivalent C^α^ positions, respectively.

## 4. Conclusion   

Taken together, we have demonstrated the utility of a PAS polypeptide as a precipitant to grow protein crystals, adding this novel class of biopolymers to the toolset for protein crystallography. Variations in length and PAS sequence as well as amino-acid composition, as described previously (Breibeck & Skerra, 2018 ▸; Schlapschy *et al.*, 2013 ▸), may offer parameters for its future optimization as a novel type of protein crystallization reagent.

## Supplementary Material

PDB reference: hen egg-white lysozyme, 6yjw

PDB reference: *O*-methyltransferase, 6yjx

## Figures and Tables

**Table 1:** Data-collection and refinement statistics Values in parentheses are for the highest resolution shell.

	HEL	FaOMT
Data collection		
Space group	*P*4_3_2_1_2	*P*2_1_2_1_2_1_
*a*, *b*, *c* (Å)	79.47, 79.47, 37.85	70.93, 89.33, 150.90
Wavelength (Å)	0.9184	0.9184
Resolution (Å)	30.0–1.20 (1.30–1.20)	35.0–2.10 (2.20–2.10)
Completeness (%)	99.8 (99.8)	99.9 (99.9)
Unique reflections	38399 (8070)	56709 (7296)
Multiplicity	8.4 (8.2)	8.9 (8.6)
Mean *I*/σ(*I*)	26.0 (2.4)	18.3 (2.5)
*R*_meas_ (%)	4.0 (97.3)	10.7 (108.0)
Wilson *B* factor (Å^2^)	19.7	38.4
Refinement		
Resolution (Å)	28.1–1.20 (1.23–1.20)	34.78–2.10 (2.15–2.10)
Reflections (working)	36485 (2661)	53735 (3942)
Reflections (test)†	1914 (129)	2974 (204)
*R*_cryst_ (%)	14.6 (68.0)	17.2 (28.1)
*R*_free_ (%)	17.5 (68.3)	20.0 (28.6)
Protein molecules per asymmetric unit	1	2
No. of atoms		
Protein	1069	5464
Ligand	—	52
Solvent‡	169	467
*B* values of atoms (Å^2^)		
Protein	16.4	35.1
Ligand	—	27.1
Solvent	30.5	40.0
Ramachandran plot§		
Favored (%)	98.4	99.6
Outliers (%)	0.0	0.0
R.m.s.d., bonds (Å)	0.01	0.01
R.m.s.d., angles (°)	1.89	1.56

† The test set corresponds to 5% of all reflections.

‡ Solvent refers to ions, ordered buffer, water and cryoprotectant molecules.

§ Ramachandran statistics were calculated with *MolProbity* (Chen *et al.*, 2010 ▸).
